# Obesity Might Be a Predictor of Weight Reduction after Smoking Cessation

**DOI:** 10.1155/2017/2504078

**Published:** 2017-08-14

**Authors:** Charlotta Pisinger, Helle Øster Nielsen, Caroline Kuhlmann, Susanne Rosthøj

**Affiliations:** ^1^Research Centre for Prevention and Health, Glostrup Hospital, Building 84/85, 2600 Glostrup, Denmark; ^2^Faculty of Health and Medical Sciences, University of Copenhagen, 1123 Copenhagen K, Denmark; ^3^Section of Biostatistics, Institute of Public Health, University of Copenhagen, 1014 Copenhagen, Denmark

## Abstract

**Background and Objectives:**

Approximately one in five ex-smokers reduces or maintains weight after smoking cessation but little is known about who succeeds to avoid weight gain. The purpose of this study was to identify predictors of weight reduction after long-term smoking cessation in a general population.

**Methods:**

Data was obtained from two Danish population-based cohorts (the Inter99 and the Helbred2006 study). Anthropometric measurements were performed by trained research staff. Out of 3.577 daily smokers at baseline 317 participants had quit smoking at the five-year follow-up for at least one year. Multiple logistic regression analysis was performed to determine predictors of weight reduction.

**Results:**

Thirteen percent reduced weight by at least 1 kg and 4% maintained their weight. Quitters with obesity had more than seven times higher odds than normal weight quitters to lose weight (OR 7.13 (95% CI 2.76–19.71)), and they had the largest median weight loss of 4.45 kg. The only other significant predictor of weight reduction was low tobacco consumption at baseline.

**Conclusions:**

Predictors of weight reduction after smoking cessation were high body mass index and low tobacco consumption at baseline. This study might motivate smokers with obesity to quit smoking and health professionals to give them support.

## 1. Introduction

Tobacco kills nearly six million people each year world-wide and unless action is taken, the annual death toll could increase to more than eight million by 2030 [1]. The benefits of smoking cessation are huge both when it comes to reduction of risk of specific diseases and to a substantial life extension [2]. Smoking cessation is one of the most cost-effective methods to prevent disease in a population [3] and it is estimated that smokers require $ 1,046 each year in additional health care expenses compared to nonsmokers [4]. Even though about three out of four smokers wish to quit [5] many smokers experience barriers when it comes to serious quit attempts. A fear of weight gain is one of the concerns reported by many smokers and weight gain is also a frequent reason given by those who relapse [6, 7].

Smokers gain weight after they quit smoking mainly because of the removal of nicotine's effects on the central nervous system [8]. Adult smokers weigh, on average, approx. 4 kg less than nonsmokers and ex-smokers gain on average the “missing” 4-5 kg after smoking cessation [9]. The mechanisms of weight gain after smoking cessation are complex and include increased appetite and energy intake, increased intake of sugar, decreased resting metabolic rate, and increased lipoprotein lipase activity [10], but the molecular mechanisms are not fully understood. Animal studies have shown that nicotine significantly decreased body weight and food intake via a decrease in meal size and a longer intermeal interval [11]. Nicotine increases energy expenditure both by direct effects on peripheral tissues, largely mediated by catecholamines, and by effects on central nervous system neuroendocrine circuits. The effect of nicotine on the brain also leads to suppression of appetite, and smoking per se (hand to mouth behaviour) can serve as an alternative to eating [12].

Limited information has been reported in relation to maintenance or reduction of weight after smoking cessation, even though up to one out of five ex-smokers actually reduce or maintain weight after smoking cessation [13]. Many studies have investigated predictors of weight gain but to our knowledge only one study has identified predictors for weight loss after smoking cessation [14]. The unilateral focus on weight gain might have contributed to the misunderstanding in the general population that weight gain is an inevitable consequence of smoking cessation.

Two more recent Danish population-based studies followed smokers over a period of five years, giving us the opportunity to explore the topic of weight reduction after smoking cessation.

The aim of this study was to identify predictors for weight reduction after long-term smoking cessation. Furthermore, we wanted to determine the proportion of ex-smokers with weight reduction or maintenance after smoking cessation and the median weight reduction over a five-year period.

## 2. Materials and Methods

### 2.1. Study Design and Participants

The study population is based on data from two population-based cohorts, the Inter99 study and the Helbred2006 study. The two studies included persons living in the south-western area of Copenhagen randomly selected from the Danish Civil Registration System and took place at Research Centre for Prevention and Health, Rigshospitalet-Glostrup. A written consent form was obtained from all participants. Both studies were approved by the Ethical Committee of Copenhagen County. The Helbred2006 study was approved by the Danish Data Protection Agency and the Inter99 study was approved by the Danish Health and Medicines Authority.

The Inter99 study is the largest randomized lifestyle intervention study in Denmark (1999–2006) including 61,301 persons aged 30–60 years. The aim of the Inter99 study was to assess the effect of a preventive strategy including individualized risk assessment; multifactorial nonpharmacological intervention based on tailored information, motivation, and support; and a programme for maintenance [15]. The subjects were prerandomized into control group (*n* = 48,285; no data on smoking or weight available) or intervention group (*n* = 13,016). Eighty-two persons had died, disappeared, or emigrated, leaving 12,934 for invitation in the intervention group. Out of these, 6,784 participants were included (inclusion = day of first examination). Participation rate (= attended the baseline visit) was 52.5%; 2,804 were daily smokers at baseline. Participation rate at 5-year follow-up visit (2004–2006) was 69.2%. The Inter99 study is described in detail elsewhere by Jorgensen et al. [15].

The aim of the Helbred2006 study (2006–2008) including 7,931 persons aged 18–69 years was to investigate prevalence and predictors of lifestyle-related chronic diseases such as coronary heart disease, diabetes, musculoskeletal disorders, asthma, allergy, chronic lung diseases, and mental disorders. Out of the almost eight thousand invited, 3,471 persons attended the baseline visit; participation rate was 44.7% (same definition as above); 773 were daily smokers at baseline. Participation rate at 5-year follow-up visit (2011) was 66.5%. Further details about the Helbred2006 cohort are described by Thuesen et al. [16].

All participants in both studies completed a questionnaire about health, lifestyle, and sociodemographic factors at inclusion. Anthropometric measurements (e.g., weight and height) performed by trained research staff were obtained.

A total of 3,577 (2,804 + 773) daily smokers were included in both studies at baseline. This paper is based on the 262 daily smokers in the Inter99 study and the 55 in the Helbred2006 study (total *n* = 317) who reported that they had quit smoking for at least 12 months at the five-year follow-up, independently of smoking status at other follow-up visits (minimal length of smoking abstinence being 12 months and maximal up to five years).

### 2.2. Variables

Weight reduction was defined as ≥1 kg lower weight at five-year follow-up than at baseline. Weight maintenance was defined as having the same or <1 kg lower weight at five-year follow-up than at baseline.

Weight gain was defined as increased weight at the five-year-follow-up compared to baseline.

Tobacco consumption was measured in grams of tobacco in the following way: one cigarette/gram of pipe tobacco = 1 gram, one cheroot/cigarillo = 3 grams, and one cigar = 5 grams. Light smokers were defined as smoking ≤15 cigarettes daily and heavy smokers >15 cigarettes daily, a common cut-point in tobacco related studies.

Height was measured without shoes to the nearest cm. Weight was measured without shoes and overcoat to the nearest kg and body mass index (BMI) was calculated (kg/m^2^). BMI was divided into three categories: normal weight (<24.9), overweight (25–29.9), and obese (>30 kg/m^2^).

Sociodemographic variables were self-reported: socioeconomic status (SES) defined by the length of completed vocational training/academic education (low: <2 years/medium: 2–4 years/high: >4 years).

Lifestyle variables were self-reported at baseline. Dietary quality score, a three-classed variable, was generated for each of the four food-groups (fish, vegetable, fruit, and fat) from a 52-item food frequency questionnaire. Summation of the four variables resulted in a score ranging from zero to eight. Subsequently participants were categorized into three classes: healthy dietary habits (score 7–9), average dietary habits score 4–6), and unhealthy dietary habits (score 1–3). The dietary quality score has previously been validated (see also the discussion section) [17]. Physical activity at baseline was based on leisure time physical activity: “During leisure time, which statement best describes your physical activity?” (read, watch TV, or be engaged in other sedentary activity/go for a walk, use your bike, or perform light physical activity for at least 4 hours a week/active athlete or performing heavy gardening, housework, etc., at least 3 times a week/vigorous training or participation in competitive sports several times a week), categorized into two groups (insufficiently active and active (light activity, moderate activity, and vigorous activity)) [18]. Alcohol consumption at baseline was calculated by adding the mean weekly intake of units of beer, strong beer, liquor, wine, and spirits in the last 12 months. Consumption was categorized to three groups (≤7, 8–14, and >14 units alcohol per week).

### 2.3. Statistical Analysis

Proportions or medians were used to describe the characteristics of the study population. Proportions were compared across subgroups using the Pearson chi-square or the Fisher exact test and the Kruskal-Wallis test was used to compare quantitative variables.

Multiple logistic regression analysis was performed to determine the associations between weight reduction and the explanatory variables. The analyses included BMI, tobacco consumption, lifestyle variables (diet, physical activity, and alcohol), sociodemographic variables (SES, sex, and age), and cohort. Possible interactions between cohort and the remaining explanatory variables were investigated, but no such interactions were found in any of the models.

To identify a set of predictive explanatory variables, a reduced model was defined using the Allen-Cady modified backwards selection procedure [19]. The explanatory variables were ranked by importance and in each step, the risk factor with the lowest rank was excluded from the model if the corresponding *p* value was below 0.05. The procedure ends when the explanatory variable with the lowest rank cannot be eliminated from the model. In this procedure the explanatory variables were ranked in the following order: BMI, tobacco consumption, lifestyle variables (diet, physical activity, and alcohol), sociodemographic variables (SES, sex, and age), and cohort.

To evaluate the discriminatory power of the models, the area (AUC) under the receiver operating characteristic curve (ROC) was determined for the logistic regression models.

To assess the robustness of the results, the analysis based on the reduced regression model was repeated using the outcome variable “no weight gain” (maintained or reduced weight).

Statistical analyses were performed using SAS version 9.2 (SAS Institute, Cary, NC, USA). The level of significance was set to 5%.

## 3. Results

Of those who were daily smokers at baseline 8.9 percent had been abstinent from smoking for at least 12 months at the five-year follow-up (317 out of 3,577). A total of 262 (82.6%) of the ex-smokers had gained weight at five-year follow-up, 41 (13%) had reduced weight by ≥1 kg, and 14 (4%) had maintained their weight.

The 41 ex-smokers who had reduced weight had a significantly higher BMI and lighter intensity of smoking at baseline than those who did not reduce weight. There were no significant differences regarding age, sex, SES, cohort, or lifestyle (Table 1). The median weight change in all ex-smokers was an increase of 5.0 kg (IQR 1.6–8.9). The median weight reduction was 3.5 kg (IQR 2.1–5.5) in those who had a weight reduction of at least 1 kg at five-year follow-up. Only tobacco consumption and cohort were associated with the size of the weight reduction, whereas BMI was borderline associated with the size of the weight reduction (*p* = 0.06) (Table 2).

In the reduced multiple logistic regression model, BMI and tobacco consumption were the only predictors of the outcome (Table 3). The effects of these two predictors were similar in the full and the reduced model.

The ROC curve analysis showed the predictive value (AUC) of the reduced model to be 0.72 (CI 95% 0.63–0.80), corresponding to a “fair” predictive value. The odds of reducing weight for heavy ex-smokers were reduced with two-thirds compared to light ex-smokers. Ex-smokers with obesity had more than seven times higher odds and overweight ex-smokers almost three times higher odds of weight reduction than those with normal weights (Table 3).

The robustness of the results analysis of the reduced logistic regression model considering “no weight gain” (weight reduction/maintenance) as outcome thereby increasing the number of cases from 41 to 55 showed attenuated effect of obesity (OR 5.03 (CI 95% 1.90–13.32)) but not of overweight (OR 2.27 (CI 95% 1.02–5.00)). Heavy smokers at baseline had less than half the chance of weight reduction/maintenance compared to heavy smokers (OR 0.42 (CI 95% 0.20–0.89)).

## 4. Discussion

This population-based study found that almost one in six persons who had quit smoking for at least one year had lost or maintained their weight. A predictor of weight reduction was a high BMI at baseline, even when adjusted for lifestyle factors and socioeconomic status. Quitters with obesity had more than seven times higher odds than normal weight quitters to lose weight. Of those of the quitters who lost weight after smoking cessation, persons with obesity at baseline had the largest median weight loss of 4.45 kg (Table 1). The only other predictor of weight reduction was low tobacco consumption at baseline, whereas baseline lifestyle factors, sex, age, and socioeconomic status, and cohort were not found to be associated with weight reduction.

A meta-analysis based on 62 studies found that 16 to 21% of ex-smokers had lost or maintained their weight [13] and a Cochrane review from 2012 found that 13% had lost weight [9]. This corresponds very well with the 13% who lost weight by ≥1 kg, and the 4% who maintained their weight in our study. The smoke-free follow-up period was 12 months in both reviews, whereas it was minimum one and up to five years in our study, so we had a longer follow-up and cessation period. For comparison, 31.4% of the whole Inter99 study population (6.784 smokers, ex-smokers, and never-smokers at baseline) had lost weight by ≥1 kg at 5-year follow-up. It is well-known that most smokers gain weight when they quit.

Predictors of weight* gain* found in previous studies have been female sex, younger age, lower socioeconomic status, and being African-American descent [8, 13, 20–22]. These factors were not significantly associated with weight loss in our study but the tendency was pointing in the same direction (ethnicity was not tested due to low number of persons with other ethnicity than Danish). Neither were cohorts, diet, or physical activity at baseline significant predictors of weight loss.

Several studies have reported a high prequit tobacco consumption or high nicotine addiction to be positively associated with weight gain [8, 23]. This supports our finding of more than 60% increased odds of weight loss in persons with low tobacco consumption.

To our knowledge, only one previous study has investigated predictors of weight* reduction* after smoking cessation [14]. This study from 1980 followed over 1700 adult males over five years and found, exactly like we did, that postcessation weight loss was associated with lighter smoking and “stoutness of build.” Strong conclusions cannot be drawn, based on two studies only, but it is interesting that a study performed more than 30 years ago concluded the same.

As previous studies have found that persons with obesity experience the largest weight gain [24, 25] it was surprising that we found that persons with obesity had the highest odds of weight reduction and the highest mean weight loss in our study. However, the findings are not irreconcilable. First of all, the studies (including ours) have used BMI (or weight) as proxy for obesity which does not distinguish between fat and musculature. The above-mentioned “stoutness of build” [14] might reflect that persons with postcessation weight loss were more muscular than fat. Second, even though most persons with obesity will gain weight some persons with obesity might be very determined not to put on additional weight after quitting smoking whereas most normal/low-weight smokers have been able to eat more or less what they desired as long as they smoked and therefore did not reduce intake of fattening food items or calories after they have quit. Also, regression towards the mean might be an attributing factor.

Unfortunately, several smoking cessation programs focusing on weight have resulted in lower quit rates [26, 27] so introducing weight-control in a smoking cessation program might actually undermine the effect of it. A Cochrane review identified exercise interventions (median −2.1 kg) and personalized weight management support (median −2.6 kg) as the only two effective treatments to prevent postcessation weight gain after one year, without undermining long-term quit rates. However, the review concluded that there are too few data to be sure [9]. It is important to have in mind that most trials aimed at preventing weight gain typically enrolled weight concerned women, which reduces their generalisability. Weight management education only, very low calorie intake, cognitive behavioural therapy to allay concern about weight gain, and pharmacotherapy for smoking cessation were not associated with long-term weight gain [9].

For many smokers, the anticipation of weight gain can hinder smoking cessation [28]. Unfortunately, we still experience overweight smokers report that health professionals have warned them not to quit, for example, after a myocardial infarction. Many health professionals have the misperception that obesity is a larger danger to health than smoking. The benefits of quitting smoking will however mostly out-weight the risks of increased weight. It has been calculated that a postcessation weight gain of almost 16 BMI units (kg/m^2^) is required for to offset the detrimental effect of continued smoking on coronary heart disease mortality. This corresponds to a weight gain of 42 kg (93 lb) for a woman 163 cm (64 in.) tall [29]. Health professionals should very early after smoking cessation detect people gaining excessive weight and intervene to prevent this by referring to exercise interventions and personalized weight management support. Results from this study need to be replicated but might hopefully motivate smokers with obesity and overweight to quit and health professionals to give them full support.

### 4.1. Weaknesses

The major weakness of this study is that out of many thousands of citizens included in two large population-based studies there were only few individuals/baseline-smokers who succeeded to quit smoking on long term and to lose weight. Results based on 41 persons must be interpreted with caution and analyses should be repeated in larger populations. Also, only a minority of persons with obesity are affected by these results.

A large weight gain might lead to smoking relapse, which would mean that those who put on large amounts of weight early in their quit attempt and relapsed were not represented by our data. People with obesity could be those with the lowest tolerance of weight gain and have high relapse rates early in the smoking cessation process, so only those who did not put on weight/lost weight are abstinent at long term. This selection-bias would highly influence our conclusions. However, no clear association between weight gain and the risk of relapse has been found [30]. We used point prevalence data in our analyses instead of continuous abstinence, but this should not affect the weight change estimates [13]. Waist circumference is found to be independently associated with cardiovascular disease, diabetes, and total mortality [31] and might have been a good alternative to BMI. Abstinence from smoking was self-reported (validation of abstinence was performed in the Inter99 study, but not the Helbred2006 study), which could lead to information-bias. This would be reflected in a lower weight gain in general but should not necessary influence our conclusions on predictors. By including only those who claimed long-term abstinence, we hope to have reduced this bias. It might be a weakness that we do not have the same length of abstinence for all participants; we measured long-term abstinence in order to look at long-term weight changes. Use of categories instead of quantitative variables imposes the risk of misclassification and reduces the power. Definition of BMI < 25 as normal weight misclassifies those who are underweight. Use of self-reported lifestyle variables might lead to underreporting of unhealthy lifestyle, especially in persons with obesity, thereby underestimating the influence of lifestyle. The dietary quality score has been shown to be associated with higher dietary quality in general, including a low intake of fat, especially saturated fat, a high intake of fiber, various vitamins and minerals, fruit, fish, vegetables, and whole-grain products and to be significantly negatively associated with total cholesterol, triglyceride, low-density lipoprotein-cholesterol, and the absolute risk of ischemic heart disease (in adjusted analyses). However, the score does not include information on sweets, sweetened beverages, or specific food as processed meet. There is a risk of underestimation of calorie intake. Physical activity at baseline was unfortunately based on leisure time physical activity only, thus underestimating energy consumption. Use of baseline lifestyle variables can be criticized as lifestyle might have changed during the long follow-up time, especially after quitting. Measuring socioeconomic status is very complex and each measurement has different strengths and weaknesses. There is no single best indicator of socioeconomic status but education has many strengths. We expected that participants in the Inter99 study, where all smokers were offered assistance to quit and all participants with obesity were offered group-based counselling to lose weight, had a higher probability of losing weight after smoking cessation. However, we did not find an association between cohort and weight reduction, which is in concordance with previous studies showing no difference in weight gain between trials that aimed to prevent weight gain after cessation and the treatment trials without this aim [5]. Defining weight loss we required a loss of 1 kg or more, to minimize measurement bias. There is no evidence for choosing this cut-off; mostly studies have only investigated if weight was higher/lower than before quitting smoking. As very few smokers had quit smoking for at least one year too few persons would have been included if the cut-off had been weight loss of, for example, 5% of body weight or more, which would be clinically more relevant. It is well-known that weight is not as stable as, for example, height, and weight cycling is rather common, especially among overweight persons, which also is a weakness in this study. The analysis of the reduced logistic regression model considering “no weight gain” (weight reduction/maintenance) as outcome thereby increasing the number of cases to 55 showed attenuated effect of obesity but not of overweight. The possibility of residual confounding due to unknown or unmeasured confounders always exists.

### 4.2. Strengths

Both cohorts were population-based and results therefore have a higher generalizability than results from randomized controlled trials on smoking cessation. We included a population of daily smokers to begin with to find the 371 persons with long-term abstinence from smoking, and compared to the existing literature we followed the cohorts for a long time. Information on weight and height was objectively measured by experienced health professionals. Analyses were consistent with or without inclusion of lifestyle- and sociodemographic factors and the cohort variable.

## 5. Conclusion

In two large Danish population-based cohorts we found that 13% had lost weight and 4% had maintained their weight after smoking cessation. A predictor of weight reduction was a high BMI, even when adjusted for lifestyle factors and socioeconomic status. Quitters with obesity had more than seven times higher odds than normal weight quitters to lose weight, and they had the largest median weight loss of 4.45 kg. The only other predictor of weight reduction was low tobacco consumption at baseline, whereas baseline lifestyle factors, sex, age, socioeconomic status, and cohort were not found to be associated with weight reduction. For many smokers, the anticipation of weight gain can hinder smoking cessation and many lay people and health professionals have the misperception that obesity is a larger danger to health than smoking. The benefits of quitting smoking will however mostly out-weight the risks of increased weight; almost 16 kg/m^2^ BMI units is required for to offset the detrimental effect of smoking. Results from this study might hopefully motivate smokers with obesity and overweight to quit and health professionals to give them full support.

## Figures and Tables

**Table 1:** Baseline characteristic of ex-smokers with long-term abstinence at five-year follow-up (*N* = 317).

Baseline variables	Weight reduction min. 1 kg		*p* value
	No (%)	Yes (%)	
	276 (87)	41 (13)	
Body mass index kg/m^2^			**<0.01**
Obese BMI ≥ 30	31 (11.2)	12 (29)	
Overweight BMI 25–29.9	107 (38.8)	20 (49)	
Normal weight BMI 18,5–24.9	138 (50.0)	9 (22)	
Tobacco consumption			**0.02**
Light smoker < 15 g tobacco/day	139 (50.6)	29 (70.7)	
Heavy smoker ≥ 15 g tobacco/day	136 (49.5)	12 (29.3)	
	*Missing n = 1*		
Cohort			^*∗*^0.17
Inter1999	228 (82.6)	34 (82.9)	
Health2006	48 (17.4)	7 (17.1)	
Socioeconomic status (length of education)			0.41
High (>4 years)	27 (10.9)	7 (17.9)	
Middle (2–4 years)	180 (72.6)	25 (65.1)	
Low (<2 years)	41 (16.5)	7 (17.9)	
	*Missing n = 30*		
Sex			0.68
Male	151 (54.7)	21 (51.2)	
Female	125 (45.3)	20 (48.8)	
Age groups			0.85
18–39 years	40 (14.5)	5 (12.2)	
40–49 years	120 (43.5)	17 (41.5)	
50–69 years	116 (42)	19 (46.3)	
Diet score			0.53
Healthy (7–9)	26 (9.5)	6 (16.6)	
Average (4–6)	201 (74.2)	30 (73.1)	
Unhealthy (1–3)	44 (16.2)	5 (12.2)	
	*Missing n = 5*		
Physical activity (leisure time)			0.84
Active	211 (77)	31 (75.6)	
Insufficiently active	63 (23)	10 (24.4)	
	*Missing n = 2*		
Alcohol units per week			0.42
>14	82 (33.3)	10 (26.3)	
8–14	56 (22.7)	7 (18.4)	
≤7	105 (42.7)	20 (52.6)	
	*Missing n = 33*		

^*∗*^Fischer's exact test.

**Table 2:** Baseline characteristics of ex-smokers at five year follow-up with a weight reduction of minimum 1 kg (*N* = 41).

Baseline variables	*N*	Weight loss (%)		Weight reduction (kg)		*p*-value
		Median (upper-lower quartile)	Mean	Median (upper-lower quartile)	Mean	
Body Mass Index kg/m^2^						0.06
Obese BMI ≥ 30	12	4.98 (2.8–10.9)	8.04	4.45 (2.6–10.9)	7.37	
Overweight BMI 25–29.9	20	5.37 (2.7–6.9)	6.08	4.15 (2.1–5.7)	4.93	
Normal weight BMI 18,5–24.9	9	3.25 (2.4–4.9)	3.61	2.30 (1.6–2.9)	2.37	
Tobacco consumption						**<0.01**
Light ≤ 15 g tobacco/day	29	3.25 (2.6–5.9)	4.59	2.50 (1.9–4.4)	3.50	
Heavy > 15 g tobacco/day	12	6.18 (4.8–14.6)	9.79	5.10 (3.7–15.4)	8.92	
Cohort						**0.04**
Inter99	34	4.96 (2.8–7.2)	6.69	3.65 (2.2–5.9)	5.61	
Health2006	7	2.92 (1.4–5.7)	3.29	1.90 (1.2–4.3)	2.53	
Socioeconomic Status (length of education)						0.24
High (>4 years)	7	7.16 (2.7–13.7)	7.49	4.70 (2.1–13.8)	7.16	
Middle (2–4 years)	25	3.59 (2.6–5.7)	5.21	3.00 (1.9–4.4)	3.99	
Low (<2 years)	7	5.73 (4.0–7.8)	7.73	4.20 (3.6–6.1)	6.69	
	*Missing n = 2*					
Sex						0.28
Male	21	4.55 (2.8–7.8)	6.44	3.70 (2.2–6.1)	5.92	
Female	20	4.76 (2.6–6.4)	5.76	2.95 (1.9–4.6)	4.21	
Age groups						0.54
18–39 years	5	4.91 (2.0–6.6)	4.72	2.50 (1.6–5.5)	3.80	
40–49 years	17	4.61 (3.5–6.6)	6.40	3.60 (3.0–5.9)	5.49	
50–69 years	19	3.25 (2.6–6.2)	6.22	2.30 (1.9–4.7)	5.06	
Diet score						0.30
Healthy (7–9)	6	3.61 (2.0–5.7)	3.73	2.40 (1.4–4.2)	2.67	
Average (4–6)	30	4.25 (2.8–6.6)	6.24	3.55 (2.1–5.8)	5.33	
Unhealthy (1–3)	5	5.00 (4.9–8.1)	8.18	4.10 (2.5–8.1)	6.54	
Physical activity (leisure time)						0.72
Active	31	3.95 (2.6–6.6)	5.85	5.20 (1.9–5.5)	4.89	
Insufficiently active	10	4.96 (3.1–8.1)	6.92	3.40 (2.3–8.1)	5.69	
Alcohol units per week						0.29
>14	10	2.90 (2.0–5.7)	4.77	2.15 (1.6–4.3)	3.65	
8–14	7	7.16 (2.7–13.7)	7.53	4.70 (2.1–13.8)	7.17	
≤7	21	4.76 (2.8–6.0)	6.38	3.50 (2.2–4.4)	5.12	
	*Missing n = 3*					
Ex-smokers with weight reduction	41	4.61 (2.6–6.6)	6.11	3.50 (2.1–5.5)	5.09	

**Table 3:** Predictors of ex-smokers' weight reduction after five years. Model 1 including all tested predictors. Model 4 including final predictors.

Baseline variables	Model 1 incl. all explanatory variables (*N* = 257)				Model 4 (*N* = 316)			
	Estimate	CI 95%	*p* value	Overall	Estimate	CI 95%	*p* value	Overall
	OR			test	OR			test
Heavy smoker	0.37	(0.16–0.86)	**0.02**	**0.02**	0.34	(0.16–0.72)	**<0.01**	**<0.01**
Light smoker (ref.)	1.00				1.00			

Obese	7.13	(2.46–20.69)	**<0.01**	**0.00**	7.38	(2.76–19.71)	**<0.01**	**<0.01**
Overweight	2.72	(1.10–6.73)	**0.03**		3.10	(1.34–7.16)	**0.01**	
Normal weight (ref.)	1.00				1.00			

Inter99	1.25	(0.43–3.65)	0.68	0.68	—	—	—	
Health2006 (ref.)	1.00							

High socioeconomic status	0.85	(0.23–3.23)	0.81	0.59	—	—	—	
Middle socioeconomic status	0.60	(0.21–1.77)	0.36		—	—	—	
Low socioeconomic status (ref.)	1.00				—	—	—	

Male	1.37	(0.59–3.18)	0.47	0.47	—	—	—	
Female (ref.)	1.00				—	—	—	

Age (years)	1.02	(0.98–1.08)	0.34	0.34	—	—	—	

Healthy diet	1.36	(0.30–6.23)	0.70	0.82	—	—	—	
Average diet	0.94	(0.28–3.22)	0.93		—	—	—	
Unhealthy diet (ref.)	1.00				—	—	—	

Active (physical activity)	0.65	(0.25–1.67)	0.37	0.38	—	—	—	
Insufficiently active (ref.)	1.00				—	—	—	

>14 alcohol units/week	0.68	(0.27–1.67)	0.40	0.47	—	—	—	
8–14 alcohol units/week	0.56	(0.29–1.59)	0.28		—	—	—	
≤7 alcohol units/week (ref.)	1.00				—	—	—	

ROC	AUC	CI 95%			AUC	CI 95%		

Predictive value	0.72	(0.63–0.80)			0.75	(0.67–0.83)		

Area under the ROC-curve (AUC).
